# Complete mitochondrial genome of the ragworm annelid *Hediste diversicolor* (of Müller, 1776) (Annelida: Nereididae)

**DOI:** 10.1080/23802359.2021.1970644

**Published:** 2021-09-06

**Authors:** André Gomes-dos-Santos, Andreas Hagemann, Luísa Valente, Arne M. Malzahn, Óscar Monroig, Elsa Froufe, L. Filipe C. Castro

**Affiliations:** a CIIMAR/CIMAR – Interdisciplinary Centre of Marine and Environmental Research, University of Porto, Porto, Portugal; b Department of Biology, Faculty of Sciences, University of Porto, Porto, Portugal; c SINTEF Ocean, Environment and New Resources, Trondheim, Norway; d ICBAS, Instituto de Ciências Biomédicas de Abel Salazar, Universidade do Porto, Porto, Portugal; e Instituto de Acuicultura Torre de la Sal (IATS-CSIC), Castellón, Spain

**Keywords:** Annelida, Nereididae, mitogenome, Lophotrochozoa

## Abstract

Marine annelids are a globally distributed and species-rich group, performing important ecological roles in macrobenthic communities. Yet, the availability of molecular resources to study these organisms is scarcer, comparatively with other phyla. Here, we present the first complete mitogenome of the Atlantic ragworm *Hediste diversicolor* (OF Muller, 1776). The mitogenome (15,904 bp long) contains 13 protein-coding genes, 22 transfer RNA, and two ribosomal RNA genes, all encoded in the same strand. Gene arrangement and composition are identical to those observed in two available congeneric species, *Hediste diadroma* and *Hediste japonica*. The phylogenetic analysis using both maximum-likelihood and Bayesian inference methods reveal a well-supported monophyly of genus *Hediste* and the already reported paraphyletic relationships within the subfamilies Nereidinae and Gymnonereidinae. Our results highlight the relevance of increasing the molecular sampling within this diverse group of marine fauna.

Annelids (∼20,000 species) are one of the most diverse group of metazoans, representing one of the three major metameric segmented animal taxa, distributed throughout marine, brackish, freshwater, and terrestrial ecosystems (Struck et al. 2011; Weigert and Bleidorn 2016). Comprehending the evolutionary history underscoring their complex body plan is fundamental for understanding Bilateria evolution (Tessmar-Raible and Arendt 2003; Raible et al. 2005; Rivera and Weisblat 2009; Struck et al. 2011). Marine annelids are distributed from deep sea to intertidal zones, playing a dominating role in macrobenthic communities (Nygren 2014; Kim et al. 2015). Moreover, they represent valuable resources for fishing as well as valuable food items in aquaculture (Pombo et al. 2020), also providing a nutritionally correct balance of polyunsaturated fatty acids (PUFAs) to crustaceans and finfish (Cardinaletti et al. 2009), a probable consequence of their endogenous capacity to *de novo* synthesize PUFAs (Kabeya et al. 2020).

Cryptic speciation events are common within marine annelids (Nygren 2014). Consequently, molecular tools represent a fundamental approach to study annelid systematics, with mitogenomes showing to be particularly useful to infer phylogenies (see examples Struck et al. 2011; Liu et al. 2012; Nygren 2014; Weigert et al. 2016; Weigert and Bleidorn 2016; Alves et al. 2020). Despite this, some annelid groups, such as the marine ragworms of family Nereididae (Blainville, 1818), are still poorly represented by complete mitochondrial genomes (Alves et al. 2020). As of April of 2021, only 23 Nereididae mitochondrial genomes were available on NCBI (i.e. 2.8% of the species described). The importance of the application of molecular data to study this family has been recently evidenced in two studies that revealed the paraphyletic status of two traditionally recognized morphological subfamilies (Nereidinae and Gymnonereidinae) (Liu et al. 2012; Alves et al. 2020). Furthermore, the use of complete mitogenomes highlighted the existence of two distinct gene orders within Nereididae, which also disagrees with the morphologically described subfamilies (Alves et al. 2020). All these highlight the importance of reevaluating morphological taxonomic assessments and increasing the availability of molecular markers for these organisms.

The genus *Hediste* (Malmgren, 1867) comprises five widely distributed Nereididae species, generally found in shallow brackish waters of the North Atlantic, East Asia, and North Pacific coastlines (Sato and Nakashima 2003; Kim et al. 2016; Park et al. 2020). Two of the five species have their complete mitogenome sequenced, i.e. *Hediste diadroma* (Sato and Nakashima 2003; Park et al. 2020) and *Hediste japonica* (Izuka, 1908) (Park et al. 2020). Producing new mitochondrial genomes will help to fully explore the evolutionary history and phylogenetic relationships within the genus, as well as within the highly diverse Nereididae family. Here, we present the first mitogenome of the Atlantic ragworm *Hediste diversicolor* (OF Muller, 1776), a species widely distributed in both sides of the temperate Atlantic (Geoffrey 2018).

An adult *H. diversicolor* specimen was collected by Andreas Hagemann in Trondheim Fjord, in Leangbukta, Norway at 63.439151 N, 10.474605 E, where the species is known to occur (e.g. Wang et al. 2019, 2020a, 2020b). A specimen was deposited at the Interdisciplinary Center of Marine and Environmental Research – CIIMAR (Prof. Filipe Castro, filipe.castro@ciimar.up.pt) under the voucher number 4HDIV3. Genomic DNA extraction was performed using a tail segment and whole-genome sequencing with Illumina 150 bp paired-end (PE) reads was performed by Novogene (Cambridge, UK). The mitogenome was obtained using a 10% subsample of the sequenced PE reads using GetOrganelle v1.7.1 (Jin et al. 2020). Annotation was performed using MITOS2 (Bernt et al. 2013). For the phylogenetic analyses, the sequenced *H. diversicolor*, all available Nereididae (*n* = 23), as well as 10 additional annelid mitogenome sequences were used. Individual alignments of the 13 protein-coding genes (PCG) were produced using GUIDANCE (v.1.5) (Sela et al. 2015), trimmed with TrimAl v.1.2 (Capella-Gutiérrez et al. 2009), and concatenated with FASconCAT-G (https://github.com/PatrickKueck/FASconCAT-G) resulting in 9630 bp. Phylogenetic inferences were conducted using maximum-likelihood (ML) in IQ-TREE v.1.6.12 (Nguyen et al. 2015) (with 10,000 ultrafast-bootstraps) and Bayesian inference (BI) in MrBayes v3.2.7 (Ronquist et al. 2012) (two independent runs of 10^7^ generations with a sampling frequency of 1000 trees). The best evolutionary models for each partition were selected in PartitionFinder v2.2.1 (Lanfear et al. 2016) for the MrBayes and by ModelFinder through IQ-TREE v.1.6.12 (Nguyen et al. 2015; Kalyaanamoorthy et al. 2017) for IQ-Tree.

The circularized *H. diversicolor* mitogenome (MW377219) has a total length of 15,904 bp, a GC content of 34.73% and encodes 13 PCGs, 22 transfer RNA, and two ribosomal RNA genes, all in the same strand. The length, gene composition, and single strand positioning are expected within family Nereididae. The gene arrangement is consistent with that previously demonstrated in two others *Hediste* species (Kim et al. 2016; Park et al. 2020).

Both ML and BI phylogenetic trees recovered the same topology with high support for almost all nodes (Figure 1). The three *Hediste* species were recovered as monophyletic and sister to a clade including two specimens of *A. succinea* with low support for both BI and ML analyses (Figure 1). This poorly supported node, as well as the paraphyly of the morphological described subfamilies Nereidinae and Gymnonereidinae are in accordance with a recent mito-phylogenetic study (Alves et al. 2020). Overall, the results obtained in the present study reinforce the importance of increasing the molecular sample representation within the family, as only then a comprehensively informed taxonomic revision will be possible.

**Figure 1.**
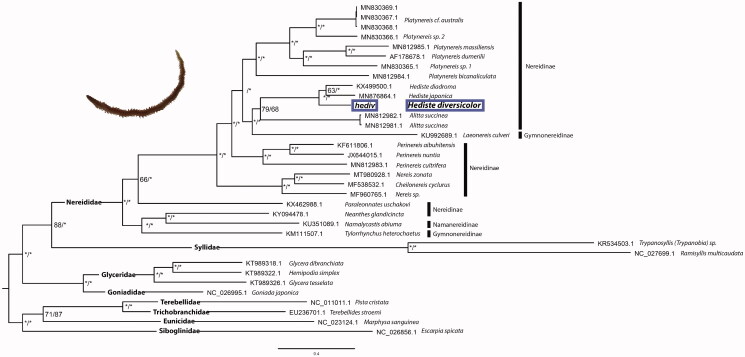
Bayesian inference phylogenetic tree based on 33 annelid sequences of 13 concatenated protein-coding genes. GenBank accession numbers are shown ahead of species names. The * above the branches indicate both posterior probabilities and bootstrap support values above 95%.

## Data Availability

The genome sequence data that support the findings of this study are openly available in GenBank of NCBI at https://www.ncbi.nlm.nih.gov under the accession no. MW377219. The associated BioProject, SRA, and Bio-Sample numbers are PRJNA737737, SRR14820308, and SAMN19707917, respectively.
